# A severe presentation of chronic non-infectious osteomyelitis associated with ulcerative colitis: a case report

**DOI:** 10.1186/s12887-020-02215-5

**Published:** 2020-06-22

**Authors:** Alyssa Lorenze, Lukas Meadows, Temitope Kehinde, Cortney Ballengee Menchini

**Affiliations:** 1 Department of Pediatrics, West Virginia University Health Sciences Center, PO Box 9214, Morgantown, WV 26506-9214 USA; 2 Department of Radiology, West Virginia University School of Medicine, PO Box 9235, 1 Medical Center Drive, Morgantown, WV 26506 USA; 3 Department of Pathology, Anatomy and Laboratory Medicine, West Virginia University School of Medicine, 64 Medical Center Drive, 2148 HSC N, Morgantown, WV 26506 USA; 4 Department of Pediatrics, Division of Pediatric Gastroenterology, Hepatology and Nutrition, West Virginia University Health Sciences Center, PO Box 9214, Morgantown, WV 26506 USA

**Keywords:** Inflammatory bowel disease, Osteomyelitis, Steroids, Chronic non-infectious osteomyelitis, Ulcerative colitis, Joint pain

## Abstract

**Background:**

Chronic Non-Infectious Osteomyelitis (CNO) is a chronic, relapsing, self-limiting inflammation of the bone. Although it is rare, CNO has been associated with inflammatory bowel disease and frequently precedes the initial diagnosis. We present a case of CNO in a patient with known ulcerative colitis in clinical remission who presented with purulent multifocal joint effusions in the setting of elevated inflammatory markers and fever suspicious for bacterial osteomyelitis.

**Case presentation:**

Our patient is a 12-year-old girl with ulcerative colitis who presented with fevers and insidious onset of joint pain at multiple sites. She had multiple joint effusions on imaging and blood and joint cultures were negative. Biopsy of the left acromion demonstrated acute and chronic osteomyelitis with areas of necrosis and granulomatous inflammation suggestive of CNO. Patient was started on high dose corticosteroids as well as methotrexate injections with marked improvement in symptoms.

**Conclusion:**

This case highlights that while purulent effusions are often indicative of bacterial osteomyelitis, the consideration of CNO in a patient with inflammatory bowel disease (IBD) with multifocal small bone involvement and negative blood cultures should be considered.

## Background

Chronic Non-Infectious Osteomyelitis (CNO) is a rare, non-infectious autoinflammatory disease of the skeletal system of unknown origin, most commonly seen in children and adolescents. It was first described by Gideon in 1972 as a sterile, subacute, chronic symmetrical osteoarthritis [1]. CNO is characterized by insidious onset of bone pain, swelling, and focal tenderness in the presence or absence of fever; however, clinical diagnosis is often challenging due to the variability and severity of symptoms. Investigation often reveals mild leukocytosis and elevation of inflammatory markers such as erythrocyte sedimentation rate (ESR) and C-reactive protein (CRP). During radiologic examination multiple lesions in a symmetrical distribution with predilection for the metaphyseal region of long bones, sternoclavicular joints, and vertebra are often seen [2].

Cultures of the lesions are sterile and unresponsive to typical antimicrobial treatment [3]. The natural course of the disease is unpredictable, often fluctuating between acute exacerbations and spontaneous remission. Jansson et al. proposed clinical criteria to aid in the diagnosis of CNO which is confirmed by two major or one major and three minor criteria. Major criteria include radiologically proven osteolytic bone lesion, multifocal bone lesions, palmoplantar pustulosis or psoriasis, and sterile bone biopsy with signs of inflammation and/or fibrosis. Minor criteria are normal blood count in a generally healthy patient, mild-moderate elevation in CRP and ESR, duration of longer than 6 months, hyperostosis, other associated autoimmune diseases, and first- or second-degree relatives with autoimmune or autoinflammatory disease [4]. Nonsteroidal anti-inflammatory drugs (NSAIDs) are considered first line treatment and are effective in most patients. In patients with known inflammatory bowel disease (IBD), other treatments including corticosteroids, methotrexate, sulfasalazine, colchicine and azithromycin must be considered to avoid inducing a flare [5]. CNO typically resolves gradually within 1–2 years of treatment [2].

The association of CNO and other autoinflammatory diseases have been reported in the literature [2, 3]. Although poorly understood, CNO is often considered a rare extraintestinal manifestation of IBD [5]. The association of CNO with ulcerative colitis (UC) is less frequently reported compared to that with Crohn’s disease (CD) [6]. CNO can often precede the symptoms of other autoinflammatory diseases such as IBD, making it important for physicians to stay attentive to new symptoms during the disease evolution.

## Case presentation

**Fig. 1:**
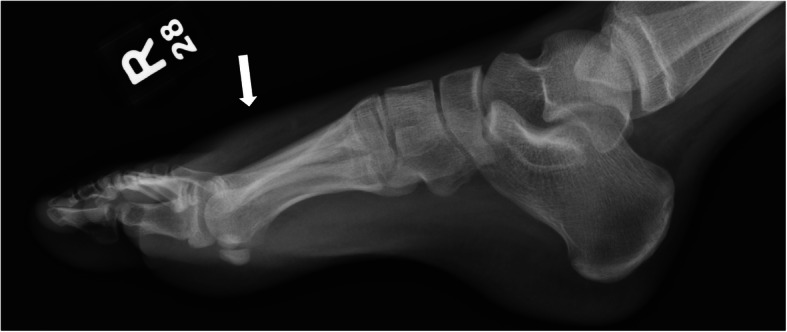
Right foot radiograph demonstrated mild soft tissue swelling and fat stranding (white arrow) which can be seen with cellulitis or edema. No definite radiographic evidence of osteomyelitis was identified

**Fig. 2:**
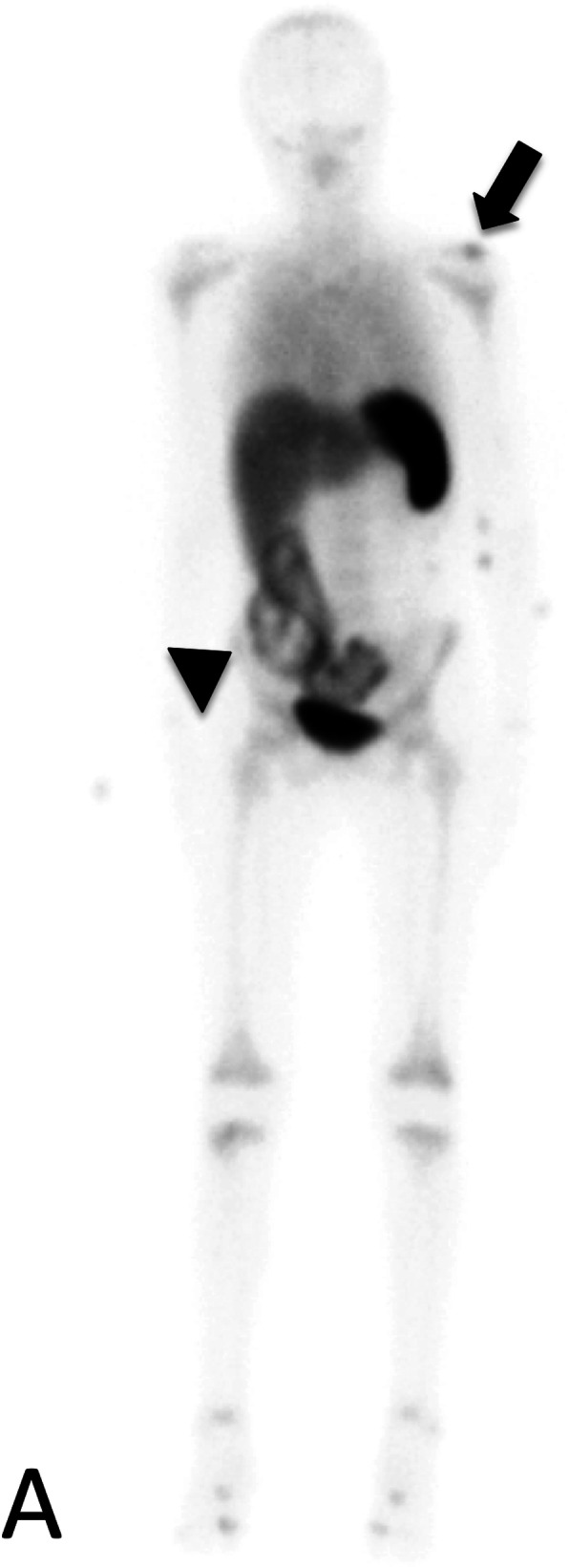
Nuclear Infection Imaging: 99 m Tc Ceretec WBC scan demonstrates abnormal focal radiotracer uptake at the left shoulder on delayed whole-body imaging (black arrow). Abnormal focal radiotracer uptake is also seen in the bowel of the right lower quadrant and pelvis, consistent with the patient’s history of ulcerative colitis (black arrowhead)

**Fig. 3:**
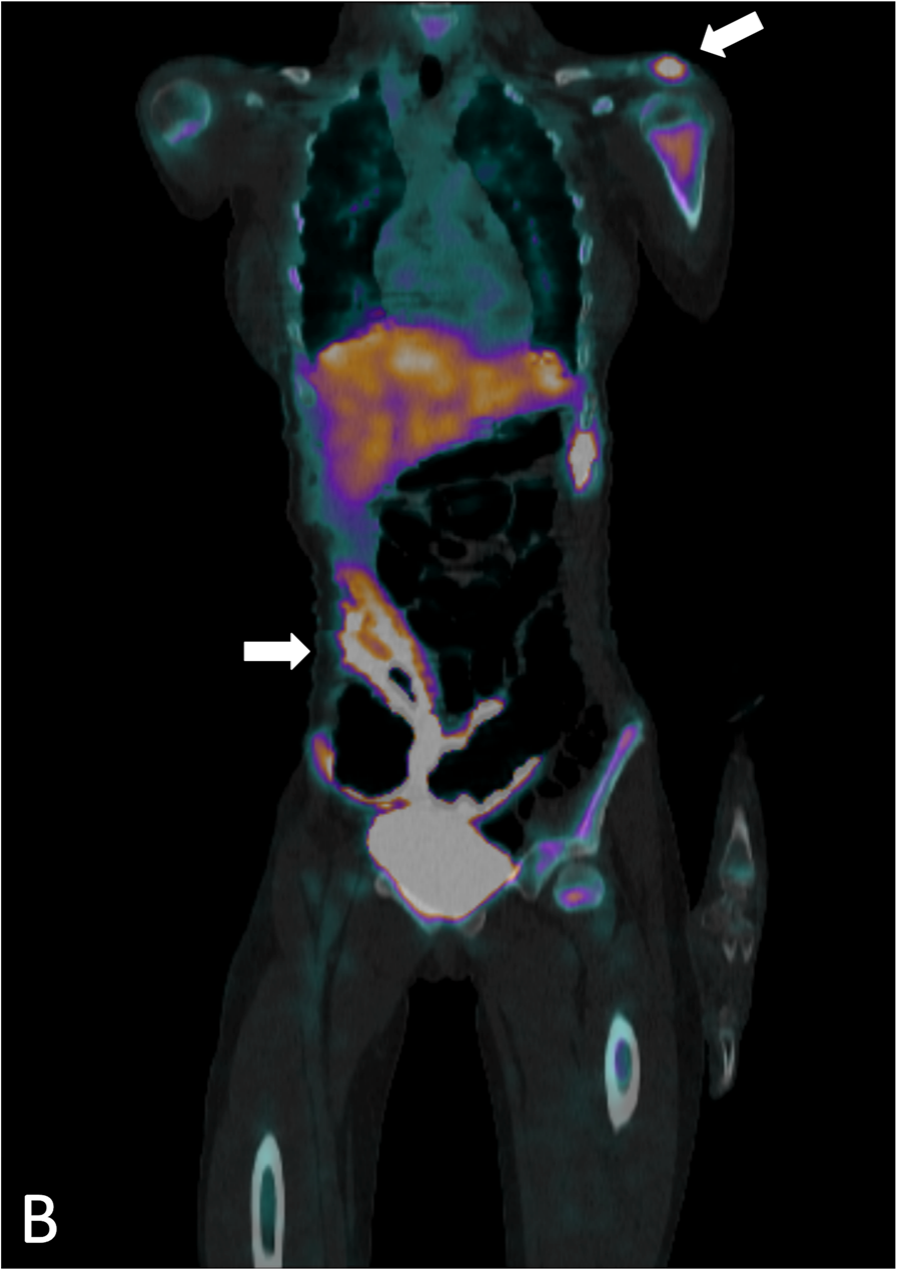
Single-photon emission computerized tomography (SPECT) scan demonstrates lytic changes at the left acromial apophysis and colon (white arrows)

**Fig. 4:**
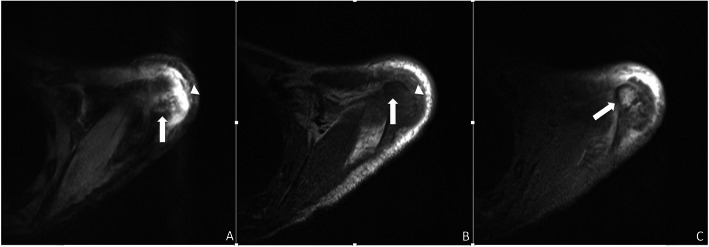
Left shoulder MRI axial images demonstrate signal characteristics consistent with osteomyelitis at the acromial apophysis: hyperintense (brighter) on T2 weighted imaging (**a**) and hypointense (darker) on T1 weighted imaging (**b**) with contrast enhancement on post-gadolinium T1 weighted imaging with fat suppression (white arrow **c**). Cortical disruption (arrowhead **b**) and an overlying fluid collection (arrowhead **a**) are also present

**Fig. 5:**
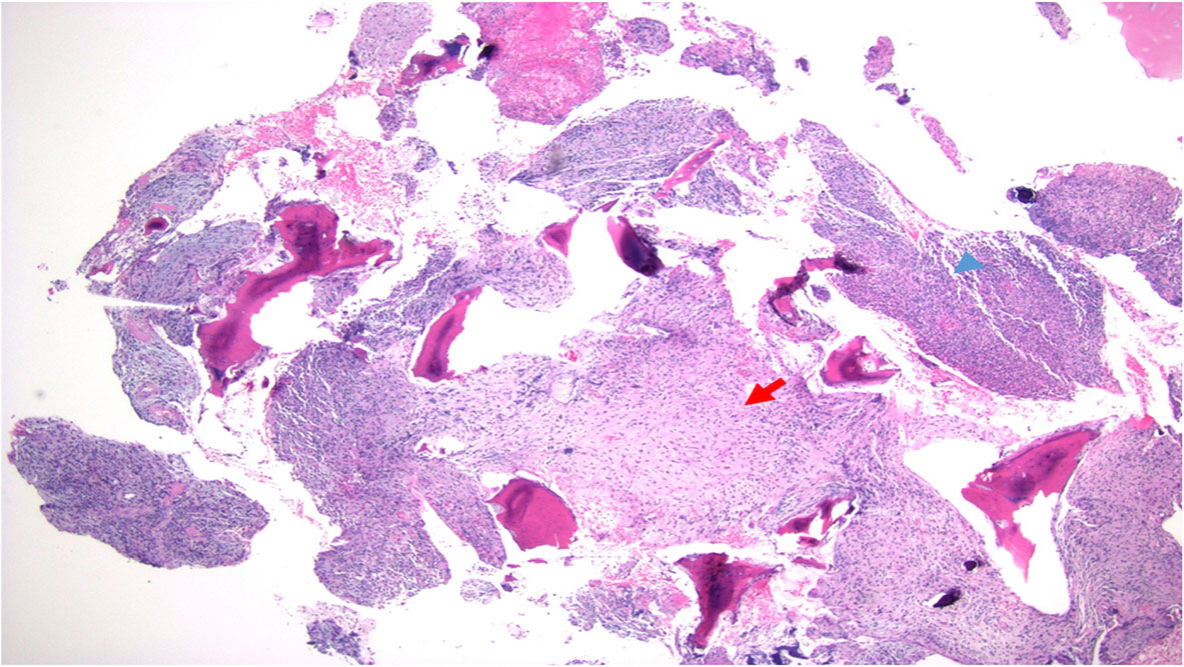
Acute and chronic osteomyelitis with areas of marrow fibrosis (red arrow) and dense lymphoplasmacytic inflammatory infiltrate (blue arrow)

**Fig. 6:**
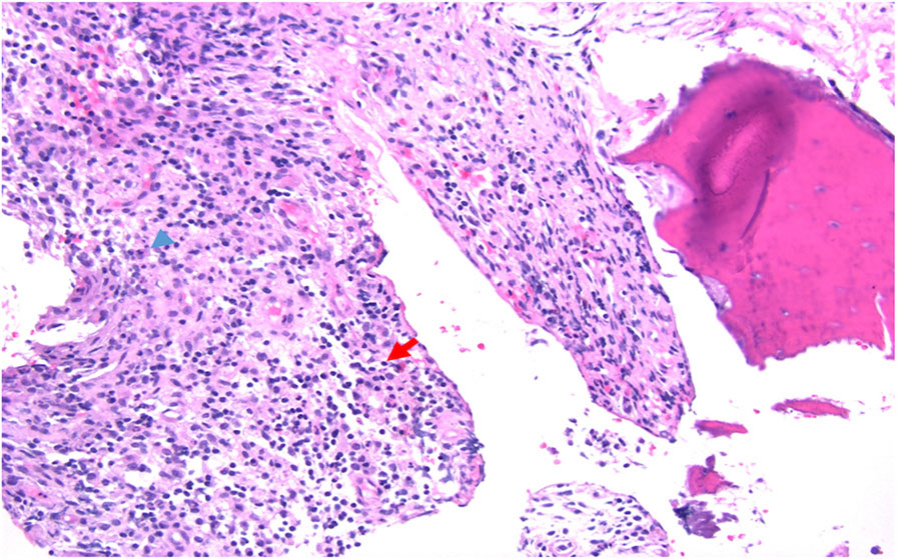
Acute and chronic osteomyelitis with dense inflammatory infiltrate. Neutrophil (blue arrowhead) and lymphocyte (red arrow)

Our patient is a 12-year-old girl with ulcerative pancolitis, diagnosed at age 10 at an outside facility. On diagnosis, she presented with a one-month history of abdominal pain, hematochezia, and a twenty-pound weight loss with a pediatric ulcerative colitis activity index (PUCAI) score of 60. Colonoscopy at initial diagnosis reported pancolitis with pathology showing chronic active colitis and crypt architecture changes. At the time of presentation to our facility, she was in clinical remission on infliximab 10 mg/kg every 4 weeks which had recently been increased secondary to an undetectable drug level. She presented with fevers and insidious onset of bone pain at multiple sites. The pain initially began a few weeks prior in her lower back and migrated to her left clavicle and bilateral metatarsals. Vital signs on admission were significant for the following: blood pressure, 100/53 mmHg; pulse rate, 124 beats/min; and body temperature, 38.9 °C. On exam, her left shoulder was exquisitely tender to palpation with overlying erythema. Additionally, her bilateral feet were tender to touch with erythema and fluctuance around her bilateral great toes. Foot radiographs demonstrated mild soft tissue swelling and fat stranding (Fig. 1). Laboratory findings included white blood cell count 9.6 × 10^3/uL with 74% PMN’s and 10% lymphocytes, hemoglobin 11.4 g/dL, platelet 413 × 10^3/uL, albumin 3.3 g/dL, INR 1.52, erythrocyte sedimentation rate (ESR) 54 mm/hr., C-reactive protein (CRP) 185.9 mg/L. Blood cultures were obtained on admission. Nuclear infection imaging demonstrated focal uptake in the left acromion concerning for osteomyelitis (Fig. 2). Single-photon emission computerized tomography (SPECT) scan also demonstrated lytic changes at the left acromial apophysis (Fig. 3). To better elucidate the anatomy of the joint, a magnetic resonance imaging (MRI) of the left shoulder was performed which also supported the diagnosis of osteomyelitis (Fig. 4). Despite this finding, antibiotics were not initiated, and patient was taken to the operating room for further exploration. Surgical incision and drainage of the left acromion and bilateral metatarsophalangeal (MTP) joints revealed copious purulence concerning for multifocal infection and abscess. Bacterial cultures and PCR were performed on the fluid from the left acromion and bilateral metatarsophalangeal joints prior to the initiation of IV Vancomycin and Ceftriaxone. Despite these interventions, the patient continued to be febrile, tachycardic and intermittently hypotensive with suboptimal pain control. Inflammatory markers continued to remain elevated. IV antibiotics were thus discontinued after 3 days. Biopsy of the left acromion was performed which revealed acute and chronic osteomyelitis with areas of necrosis and granulomatous inflammation (Figs. 5 & 6). Aerobic, anaerobic, fungal and acid-fast bacteria (AFB) cultures and pan-bacterial PCR were negative from the joint fluid prior to the start of antibiotics eluding to a non-infectious cause. Methylprednisolone 1000 mg IV daily was initiated inpatient for a three-day course per Rheumatology recommendations with marked improvement in fever, pain and inflammatory markers. Patient was discharged on Prednisone 30 mg twice daily and started on Methotrexate 20 mg SQ weekly to be further managed by Rheumatology. The patient was seen in Rheumatology clinic 2 weeks following discharge and continued to remain afebrile and asymptomatic. She was followed monthly in Rheumatology clinic and was tapered off of steroids 5 months following her initial presentation. She has remained on Methotrexate 20 mg SQ weekly. She continued to have complete resolution of joint symptoms off of steroids without any limitations in joint movements. Furthermore, patient has followed closely with Pediatric Gastroenterology where she continues to remain in clinical remission on infliximab 10 mg/kg every 4 weeks. A colonoscopy was repeated 8 months following discharge from the hospital which showed mild proctitis.

In this case, despite the negative tests for microorganisms, the possibility of an infection cannot completely be ruled out. However, when considering that the inflammation occurred in multiple bony sites without involvement of other organs, the negative response to broad-spectrum antibiotics along with negative blood and joint fluid cultures and PCR, these clinical findings and laboratory results indicate the likelihood that the inflammation was due to a non-infectious origin.

## Discussion and conclusions

Chronic Non-Infectious Osteomyelitis (CNO) is considered a diagnosis of exclusion, often resulting in delayed symptom recognition and treatment. Diagnosis is typically based on history, physical exam, and radiological and histological findings. Our patient displayed three of the major criteria proposed by Jansson et al. based on MRI findings, multifocal bone lesions, and sterile bone biopsy with signs of inflammation.

The association between IBD and CNO remains poorly understood with only twenty-seven cases reported in the literature thus far. Of those patients, only seven of the twenty-seven carried the diagnosis of UC [5]. Multiple reports in the literature speculate that a common enticing agent could potentially lead to inflammation in both the bones and the intestines [7].

Our patient had multiple characteristics that distinguished her case from prior CNO cases associated with IBD. First, CNO occurred 2 years after ulcerative colitis was diagnosed. In most reported cases, symptoms of bone inflammation precede those of bowel symptoms by as much as 5 years. In 66% of previously reported cases, symptoms of CNO predated the onset of IBD symptoms with a median interval of 3.5 years [5]. With the etiology of both conditions being unclear, many reports have proposed a genetic predisposition to both CNO and IBD. Additionally, studies have suggested that cytokines released from the inflamed bowel could medicate joint and bone inflammation [8]. In our case, cytokine release from the bowel could have mediated to the patients affected areas causing the bony lesions.

Second, our patient was in clinical remission on biologic therapy when her bone symptoms presented. Prior reports have associated anti-tumor necrosis factor treatment with improvement in bone symptoms [5]. According to a literature review by Audu et al. only two out of twenty-four cases of patients with IBD were on anti-TNF agents at the time of CNO diagnosis [5]. Given patients undetectable anti-TNF level, it could be postulated that patients drug level was too low to even have an effect on her bone symptoms. Third, CNO is characterized by multifocal non-pyogenic, sterile, bone lesions. Our case describes a patient with known ulcerative colitis who presented with purulent multifocal joint effusions in the setting of elevated inflammatory markers and fever suspicious for bacterial osteomyelitis. While rare and underdiagnosed, clues towards CNO in our patient included chronic bone pain, culture negative biopsy off of antimicrobial therapy, shoulder pain, and a prior history of a systemic inflammatory disease. Only after further investigation, negative bacterial culture and PCR, and worsening clinical symptoms on antimicrobial therapy, was the diagnosis of CNO considered. Although the possibility of an infection cannot completely be ruled out in this case, the clinical and laboratory results as well as complete resolution of symptoms with immunosuppressive therapy highly suggest a non-infectious etiology. In addition, the patient was followed closely after discharge and continued to remain asymptomatic without disease recurrence. This case highlights that, while purulent effusions are often indicative of bacterial osteomyelitis, the association of CNO in a patient with IBD should be considered.

## Data Availability

Data sharing is not applicable to this article as no datasets were generated or analyzed during the current study.

## Abbreviations

CNO: Chronic Non-Infectious Osteomyelitis
IBD: Inflammatory Bowel Disease
UC: Ulcerative colitis
PUCAI: Pediatric ulcerative colitis activity index
WBC: White blood cell
ESR: Erythrocyte sedimentation rate
CRP: C-reactive protein
NSAIDs: Nonsteroidal anti-inflammatory drugs
MRI: Magnetic resonance imaging
MTP: Metatarsophalangeal
AFB: Acid-fast bacteria
PCR: Polymerase chain reaction
IV: Intravenous
SQ: Subcutaneous
SPECT: Single-photon emission computerized tomography
